# Physical Inactivity Is Correlated with Levels of Quantitative C-reactive Protein in Serum, Independent of Obesity: Results of the National Surveillance of Risk Factors of Non-communicable Diseases in Iran

**DOI:** 10.3329/jhpn.v30i1.11278

**Published:** 2012-03

**Authors:** Alireza Esteghamati, Afsaneh Morteza, Omid Khalilzadeh, Mehdi Anvari, Sina Noshad, Ali Zandieh, Manouchehr Nakhjavani

**Affiliations:** Endocrinology and Metabolism Research Center, Vali-Asr Hospital, School of Medicine, Tehran University of Medical Sciences, Tehran, Iran

**Keywords:** Cardiovascular diseases, C-reactive protein, Physical activity, Physical exercise, Risk factors, Iran

## Abstract

Increased C-reactive protein (CRP) levels are associated with coronary heart disease, stroke, and mortality. Physical activity prevents cardiovascular disorders, which can be partly mediated through reducing inflammation, including serum CRP levels. The association of different intensities of physical activity, sedentary behaviours, and C-reactive protein (CRP) levels in serum was examined after adjustment for markers of adiposity, including waist-circumference and body mass index (BMI), in a large population-based study. Using data of the SuRFNCD-2007 study, a large national representative population-based study in Iran, the relationship between quantitative CRP concentrations in serum and physical activity was examined in a sample of 3,001 Iranian adults. The global physical activity questionnaire (GPAQ) was used for evaluating the duration and intensity of physical activity. Total physical activity (TPA) was calculated using metabolic equivalents for the intensity of physical activity. Quantitative CRP concentrations in serum were measured with high-sensitivity enzyme immunoassay. The CRP levels in serum significantly correlated with TPA (r=-0.103, p=0.021 in men and r=-0.114, p=0.017 in women), duration of vigorous-intensity activity (r=-0.122, p=0.019 in men and r=-0.109, p=0.026 in women), duration of moderate-intensity activity (r=-0.107, p=0.031 in men and r=-0.118, p=0.020 in women), and duration of sedentary behaviours (r=0.092, p=0.029 in men and r=0.101, p=0.022 in women) after multiple adjustments for age, area of residence, BMI, waist-circumference, smoking, and diabetes mellitus. Physical activity (of both moderate and vigorous intensity) is inversely associated with the quantitative CRP levels in serum, independent of diabetes and body adiposity.

## INTRODUCTION

Adipose tissue in obesity, diabetes, and metabolic syndrome is in a state of chronic inflammation (1-5). Quantitative C-reactive protein (CRP), an acute-phase reactant produced dominantly by liver, is a plasma protein that circulates in increased amounts during inflammation and often damages the tissue (6,7). Results of studies showed that the increased CRP levels were associated with functional impairment, coronary heart disease, stroke, and mortality (8). So, reducing the CRP levels can reduce mortality and morbidity due to cardiovascular disorders.

Physical activity can prevent cardiovascular disorders (9). This effect can be partly mediated through reducing inflammation, including serum CRP levels (10). Results of some population-based studies showed a negative association between physical activity and the circulating levels of CRP (7,10-14). However, the pattern of this association with res-pect to different intensities and metabolic equivalents (METs) of physical activity and several tertiary factors, such as adiposity and glycaemic status, is not well-understood. Results of some studies showed that the impact of physical activity is mediated through its weight-lowering effect (15,16). A study reported that the circulating levels of CRP can be markedly suppressed, independent of total adiposity or fat mass, by intense regular physical exercise (17).

The present study was carried out to examine the association among different intensities of physical activity (moderate and severe), total physical activi-ty (using METs for the intensity of activities), duration of sedentary behaviours, and the serum CRP levels after adjustment for glycaemic status and markers of adiposity, including waist-circumference and body mass index (BMI) in a large population-based sample of Iranian adults. Physical activity was defined by an international standard questionnaire, named global physical activity questionnaire (GPAQ).

## MATERIALS AND METHODS

### Participants

The study was based on data collected in the third national surveillance of risk factors of non-communicable diseases (SuRFNCD-2007). Details of the survey were reported elsewhere (18). In brief, a cluster-sampling scheme was applied to randomly select a representative sample of Iranian adults aged 25-64 years. The number of clusters selected from each province was proportional to the urban/rural size of that province. Trained healthcare professionals conducted household interviews and physical examinations. All interviews were conducted in Persian. Data were recorded in standardized sets of questionnaire. Blood sampling was done within a few days of the interview.

The survey received ethical approval from the Center for Disease Control of Iran, and written informed consent was obtained from all the participants.

### Assessment of physical activity

The second version of the GPAQ was used in the survey (19). This questionnaire, developed by the World Health Organization (WHO), contains 16 questions about physical activity in a typical week and assesses physical activity in three domains: work, transportation, and recreational activities. The evaluation of physical activity in these domains is one of the factors that make the GPAQ distinct from other sets of questionnaire, such as the less-sophisticated, short version of the international physical activity questionnaire (IPAQ) (http://www.ipaq.ki.se). It also determines the intensity of activity (i.e. vigorous or moderate) in each domain and the time spent on sedentary behaviours, such as watching TV. Sedentary behaviours were defined as activities, such as sitting at a desk, travelling in car/bus/train, reading, working with computer, and watching television.

To measure energy expenditure, the concept of METs was used (19). MET is the ratio of a person's working metabolic rate and the resting metabolic rate (19). One MET is defined as the energy cost of sitting quietly and is equivalent to a caloric consumption of 1 kcal/kg/hour. It is estimated that a person's caloric consumption is four times high when moderately active and eight times high when vigorously active. Therefore, to calculate a person's overall energy expenditure, four METs are assigned to the time spent on moderate activities, and eight METs are assigned to the time spent on vigorous activities. The total physical activity (TPA) score was calculated as the sum of all METs × minutes for moderate or vigorous-intensity physical activity performed in work, commuting, and recreation.

Based on the GPAQ analysis framework (19), our participants were classified into three groups of high, moderate and low physical activity categories. More details on these definitions were reported elsewhere (20,21).

### Physical examination and biochemical measurements

Weight and height were measured in light clothing and without shoes. A portable calibrated electronic weighing scale (Omron Corp., Tokyo, Japan) and portable measuring inflexible bars (Seca, Hamburg, Germany) were used for this purpose. A constant tension tape (Seca, Hamburg, Germany) was used for measuring waist-circumference at the end of a normal expiration, with arms relaxed at the sides, at the midpoint between the lower margin of the lowest rib and the highest point of the hip on the mid-axillary line. The BMI (kg/m^2^) was calculated according to the Quetelet formula. Five mL of venous blood was taken in sitting position, centrifuged, and transferred under cold-chain condition to the laboratory. Quantitative CRP concentrations in serum were measured in the endocrine laboratory of the Vali-Asr Hospital (Tehran University of Medical Sciences) using the quantitative CRP kit (Parsazmoon, Karaj, Iran), with an intra-assay coefficient of variation of 2.6%. Diabetes was diagnosed following the criteria of the American Diabetes Association as described in our previous report (22).

### Statistical analysis

Complex analysis of the sample survey was performed using the SPSS software for Windows (version 17) (Chicago, IL, USA). Data were weighted for sex, age, and residential area (urban/rural) strata, according to the population of Iran (national census, 2006). Kolmogorov Smirnov analysis was employed to study the normality of variables. CRP concentrations in serum were log-transformed to change the distribution to normal; the log-transformed values of CRP were used in analyses. Continuous variables were expressed as mean±standard error of mean (SEM). Partial correlation coefficients were calculated between quantitative CRP and features of physical activity after adjustment for various variables. The adjusted values of quantitative CRP were compared between the categories of physical activity, using the general linear modelling method. The p value of <0.05 was considered significant.

## RESULTS

After excluding the participants with missing data in laboratory results (n=396), analyses were performed for the remaining 3,001 individuals. The demographic data (age, sex, and residential area) of the excluded subjects were not different from the remaining participants. Table 1 shows the baseline characteristics of the study participants. Forty-six percent of men were in the category of high physical activity while 40% of women fell into the cate-gory of low physical activity. There was no significant difference in the serum CRP levels between men and women.

**Table 1.** Characteristics of study participants (SuRFNCD-2007, Iran)

Characteristics	Male (n=1,494)	Female (n=1,507)	Total (n=3,001)
Age (year)*	39.42±0.75	39.76±0.78	39.59±0.54
Area of residence			
Urban (%)	1,011 (67.7)	1,004 (66.6)	2,015 (67.1)
Rural (%)	483 (32.3)	503 (33.4)	986 (32.9)
BMI (kg/m^2^)*	25.4±0.2	27.6±0.2	26.5±0.2
Waist-circumference (cm)*	88.6±0.4	88.8±0.6	88.7±0.4
Physical activity category			
Low (%)	31.6±1.1	48.6±1.3	40.0±1.0
Moderate (%)	22.3±0.1	27.1±1.2	24.7±0.8
High (%)	46.1±1.3	24.3±1.2	35.4±1.1
Total physical activity (MET-min/day)	909.2±47.3	327.9±13.8	623.2±31.9
Duration of vigorous activity (min/day)	58.1±5.7	4.9±0.9	31.9±3.9
Duration of moderate activity (min/day)	111.1±4.4	72.2±2.3	91.9±3.2
Duration of sedentary behaviours (min/day)	240.2±4.2	238.0±4.6	239.2±3.8
Current smoking (%±SE)	26.1±1.6	1.8±0.3	14.1±1.1
Diabetes mellitus	6.6±0.4	9.1±0.4	7.8±0.4
Quantitative CRP (mg/L)*	5.6±0.1	5.6±0.1	5.6±0.1

Variables, except age, sex, and area of residence, were standardized for age, sex, and residential area of the 2006 population of Iran.

*Mean±SE;

BMI=Body mass index;

CRP=C-reactive protein;

MET=Metabolic equivalent;

min=Minute;

SE=Standard error

**Table 2.** Association between several features of physical activity and serum quantitative CRP after multiple adjustments (SuRFNCD-2007, Iran)

Total physical activity (MET-min/day)	Male		Female	
	r	p	r	p
Adjustment for				
Age and area of residence	-0.127	0.011	-0.129	0.010
Age, area of residence, BMI, WC, and smoking	-0.103	0.021	-0.114	0.017
Age, area of residence, BMI, WC, smoking, diabetes mellitus, and duration of sedentary behaviours	-0.084	0.035	-0.104	0.030
Duration of vigorous activity (min/day)				
Adjustment for				
Age and area of residence	-0.131	0.008	-0.117	0.021
Age, area of residence, BMI, WC, and smoking	-0.122	0.019	-0.109	0.026
Age, area of residence, BMI, WC, smoking, diabetes mellitus, duration of moderate activity, and sedentary behaviours	-0.116	0.027	-0.106	0.029
Duration of moderate activity (min/day)				
Adjustment for				
Age and area of residence	-0.123	0.018	-0.125	0.012
Age, area of residence, BMI, WC, and smoking	-0.107	0.031	-0.118	0.020
Age, area of residence, BMI, WC, smoking, diabetes mellitus, duration of vigorous activity, and sedentary behaviours	-0.071	0.052	-0.102	0.032
Duration of sedentary behaviours (min/day)				
Adjustment for				
Age and area of residence	0.108	0.013	0.119	0.008
Age, area of residence, BMI, WC, and smoking	0.092	0.029	0.101	0.022
Age, area of residence, BMI, WC, smoking, diabetes mellitus, and TPA	0.081	0.041	0.078	0.047

BMI=Body mass index;

CRP=C-reactive protein;

MET=Metabolic equivalent;

min=Minute; SE=Standard error;

TPA=Total physical activity;

WC=Waist-circumference

The serum CRP levels correlated with age (r=0.058, p=0.024 in men and r=0.065, p=0.44 in women), BMI (r=0.029, p=0.026 in men and r=0.030, p=0.023 in women), and waist-circumference (r=0.59, p=0.021 in men and r=0.047, p=0.046 in women). Current smokers had higher levels of quantitative CRP vs ex-smokers or never-smokers (5.64±3.06 vs 5.19±2.73, p=0.139 in men and 5.60±2.68 vs 5.41±2.93, p=0.69 in women). TPA was inversely associated with age (r=-0.212, p=p.002 in men and r=-0.173, p=0.005 in women), BMI (r=-0.338, p<0.001 in men and r=-0.198, p=0.001 in women), and waist-circumference (r=-0.357, p<0.001 in men and r=-0.239, p<0.001 in women).

CRP significantly correlated with TPA (r=-0.103, p=0.021 in men and r=-0.114, p=0.017 in women), duration of vigorous-intensity activity (r=-0.122, p=0.019 in men and r=-0.109, p=0.026 in women), duration of moderate-intensity activity (r=-0.107, p=0.031 in men and r=-0.118, p=0.020 in women), and duration of sedentary behaviours (r=0.092, p=0.029 in men and r=0.101, p=0.022 in women) after multiple adjustments for age, area of residence, BMI, waist-circumference, smoking, and diabetes mellitus using partial correlation (Table 2). The correlation between quantitative CRP and TPA was independent of the duration of sedentary behaviours (r=-0.084, p=0.035 in men and r=-0.104, p=0.030 in women) (Table 2).

Considering the different categories of physical activity, there was a significant inverse correlation between quantitative CRP and higher physical activity in both men and women after multiple adjustments for age, residential area, BMI, waist-circumference, smoking, and diabetes mellitus (Fig.).

Serum CRP concentrations of ≥10 mg/L are frequently observed in subjects with inflammatory conditions (23); therefore, in a separate analysis, we excluded these patients (n=70) and repeated the association analyses once more. This change could not considerably change the level of associations (r) in different models.

## DISCUSSION

In this study, the duration and the intensity (vigo-rous and moderate) of physical activity in three domains of work, transportation, and leisure time were evaluated using the GPAQ. Our findings clearly demonstrated that physical activities of both moderate and vigorous intensity were associated with lower levels of quantitative CRP. Furthermore, we showed that this correlation was independent of several potential confounders, including BMI, waist-circumference, smoking status, diabetes mellitus, age, and sex. Note that our study, in a large population-based sample, could show a mild but significant and independent inverse association between physical activity and CRP. The mild degree of this association is expected because, in a general population, the level of CRP does not have a large variation, and the majority of people have normal ranges of CRP. However, our findings are important because it shows that, even in a general sample of a population where most people have a normal range of CRP, the level of physical activity is an important indicator, which contributes to the mild differences of CRP levels among different individuals of the population.

**Fig.**
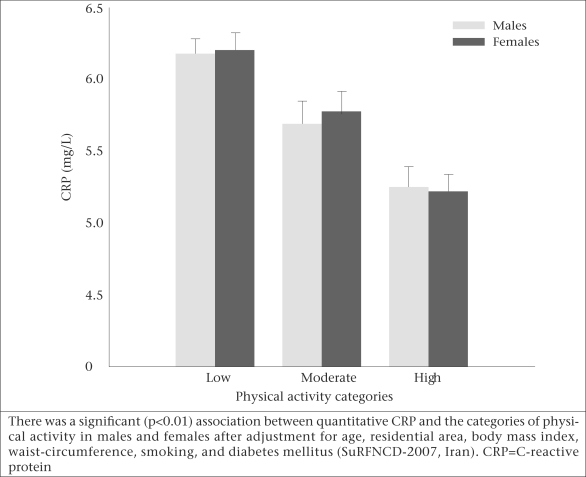
Serum CRP levels in different levels of physical activity, stratified according to gender

Our results are generally consistent with those of the growing number of studies, suggesting that high levels of physical activity are associated with lower levels of quantitative CRP (7,10). In a study which evaluated the effect of physical activity on serum CRP levels among 3,075 men and women, higher levels of exercise were associated with lower levels of quantitative CRP (11). In a cohort of 5,888 men and women aged over 65 years, those in the highest quartile of physical activity had 19% lower concentrations of quantitative CRP compared to persons in the lowest quartile (12). In another study, physical activity was significantly and inversely associated with several inflammatory variables, including quantitative CRP (13). They also showed that, after a 20-year follow-up, the quantitative CRP levels of those who were inactive at first but later took up light physical activity reached the CRP levels of the participants who were active throughout the follow-up (13). Physical activity was independently associated with lower odds of having an elevated level of quantitative CRP in the Third National Health and Nutrition Examination Survey (14). Compared to these studies, our study has the advantage of evaluating the impact of different intensities of physical activity using the concept of METs. Furthermore, our study could show that this association was independent of indicators of body adiposity and diabetes. Our findings also, for the first time, point to a significant association between the time spent on sedentary behaviour and the quantitative CRP levels, independent of physical activity and obesity.

Consistent with our findings, Fischer and colleagues showed that the plasma levels of CRP were associated with physical inactivity, independent of obesity (24). Similarly, Tomaszewski and colleagues showed that the circulating levels of CRP could be markedly suppressed, independent of total adiposity or indeed fat mass, by intense regular physical exercise (17). However, they had a small sample-size to conclude this finding. Why does physical activity reduce the serum CRP levels? Various theories have been postulated to explain this (25-27). The serum levels of TNF-α and IL-6 were reduced by physical activity, independent of the adipose tissue content (27). TNF-α and IL-6 induce the production and secretion of CRP (28). So, this may reduce the serum CRP levels, irrespective of fat content. On the other hand, physical activity induces anti-inflammatory cytokines, including IL-1 receptor antagonist and IL-10, which are known to hamper the production of CRP (26,27). We did not measure the levels of IL-6 and TNF-α in serum in our participants. Nevertheless, we encourage other researchers to evaluate the serum level of different cytokines in parallel with CRP and physical activity in the future epidemiologic studies.

Previous studies gave different weights to the health effects of moderate and vigorous physical activity (29). Some authors suggest that vigorous activity is much stronger in improving cardiovascular health but others argue that moderate activity (which can be tolerated longer in an ordinary man) can be as effective as vigorous activity (30). Our results showed that activities of both vigorous and moderate intensity were independently associated with the lower CRP level but the degree of association was higher for vigorous vs moderate in both males and females. Therefore, our study provides a support for the beneficial consequences of activities of both vigorous and moderate intensity.

### Limitations

The principal limitation of the present study is its cross-sectional nature, which precludes the determination of the direction of causality. This study was conducted in accordance with the stepwise guidelines of WHO, and our questionnaire did not gather data on history of cardiovascular diseases, malignancy, or other acute/chronic diseases (22). The decision regarding non-inclusion of history of cardiovascular diseases in the SuRFNCD questionnaire comes from the fact that there is no readily-available technique to diagnose these conditions in the cases of negative history. For example, doing exercise testing or angiography on a national scale is not feasible. Therefore, the influence of these factors was not assessed in our study; however, we believe that this limitation does not lessen the importance of our findings to show a significant crude association between the CRP levels and physical inactivity in a general population. Also, factors, such as diet or alcohol drinking status, may also be influential. Nevertheless, drinking of alcohol is illegal in Iran, and based on our experience, a very low percentage of our population was drinkers.

The principal advantage of the present study is its large sample-size, and the point that it is representative of the Iranian population. Moreover, we used a standardized international questionnaire to study the different domains of physical activity and sedentary behaviours. We also evaluated the potential confounding effects of several tertiary factors on the association between physical activity and quantitative CRP.

### Conclusions

In this study, we evaluated the association between different intensities of physical activity and the serum CRP level. We showed that both vigorous and moderate physical activities were associated with the lower serum CRP levels. This association was independent of diabetes and body adiposity. We also showed that the time spent in sedentary behaviours positively correlated with the quantitative CRP levels, independent of physical activity and obesity. The practical message of the study is that any type of physical activity (vigorous or moderate) is associated with lower CRP and can, therefore, be beneficial for a healthier lifestyle. Low activity and sedentary behaviours are independently associated with high CRP and can potentially cause negative health consequences.

## ACKNOWLEDGEMENTS

The study was supported by the Ministry of Health and Medical Education, Tehran, Iran.
